# Ratio-Based Pulse Shape Discrimination: Analytic Results for Gaussian and Poisson Noise Models

**DOI:** 10.6028/jres.126.032

**Published:** 2021-11-09

**Authors:** Kevin J Coakley

**Affiliations:** 1 National Institute of Standards and Technology, Boulder, CO 80305, USA

**Keywords:** Bayesian analysis, classification, Gaussian processes, Poisson processes, prompt fraction statistic, pulse shape discrimination, receiver-operating-characteristic curve

## Abstract

In experiments in a range of fields including fast neutron spectroscopy and astroparticle physics, one can discriminate events of interest from background events based on the shapes of electronic pulses produced by energy deposits in a detector. Here, I focus on a well-known pulse shape discrimination method based on the ratio of the temporal integral of the pulse over an early interval X_p_ and the temporal integral over the entire pulse X_t_. For both event classes, for both a Gaussian noise model and a Poisson noise model, I present analytic expressions for the conditional distribution of X_p_ given knowledge of the observed value of X_t_ and a scaled energy deposit corresponding to the product of the full energy deposit and a relative yield factor. I assume that the energy-dependent theoretical prompt fraction for both classes are known exactly. With a Bayesian approach that accounts for imperfect knowledge of the scaled energy deposit, I determine the posterior mean background acceptance probability given the target signal acceptance probability as a function of the observed value of X_t_. My method enables one to determine receiver-operating-characteristic curves by numerical integration rather than by Monte Carlo simulation for these two noise models.

## 1 Introduction

In a variety of experiments in fields such as astroparticle physics (for example, see Refs. [1–6]), fast neutron spectroscopy [7–10], and neutrino physics (for example, see Refs. [11–13]), events of interest and background events deposit energy in detectors. Typically, the shapes of measured electronic pulses generated by events of interest and background events are different. There are many different methods [14] for pulse shape discrimination (PSD), including those based on: ratios of pulse integrals corresponding to different time intervals [8, 15, com]parison to reference templates [16–18], machine learning [19–25], pulse gradient methods [26], zero-crossing analysis [27, 28], frequency gradient analysis [29], and Fourier transform analysis [30]. Here, I focus on a “prompt fraction” discrimination statistic *F_p_* defined as


(1) $$F_{p}=\frac{X_{p}}{X_{t}},$$

where *X_p_* is the integrated pulse in a prompt time interval [*T_begin_, T_prompt_* ], and *X_t_* is the integrated pulse in a total time interval [*T_begin_, T_end_* ]. I consider two cases. In one case, *X_p_* and *X_t_* are correlated Gaussian random variables. In the other case, *X_p_* and *X_t_* are correlated Poisson random variables.

There are exact and nearly exact approximations for the distribution of the ratio of Gaussian (normal) random variables with known means, variances, and correlation [31–33]. Based on Refs. [31, 32], the work in Ref. [34] includes a model to predict the distribution of prompt fraction statistics produced by a given energy deposit where the observed values of *X_p_* and *X_t_* are unconstrained. In applications of interest, data generated by a continuum of energy deposits are binned according to observed values of *X_t_* . Thus, the conditional distribution of *X_p_* given the measured value of *X_t_* is of primary interest for PSD studies and the focus of this work.

In many experimental studies, only a fraction of the energy deposited in a detector produces a measurement of interest. In general, this fraction varies for the two event classes. In this work, for each class, I assume that this fraction does not vary from event-to-event. Based on these fractions, I assign a relative yield factor *β* to each class. For the class with the higher fraction, *β* = 1. For the other class, 0 *< β ≤* 1. If both classes have the same fraction, *β* = 1 for both classes. Given the full energy deposit *e_dep_* and *β*, I define a scaled energy deposit *e* as:


(2) *e* = *β e_dep_.*

For any scaled energy deposit *e*, I assume that the expected value of *X_t_* is the same for both event classes.

For Gaussian and Poisson noise models, I derive exact expressions for the conditional distributions of *X_p_* given the measured value of *X_t_* and the unobserved value of *e*. The major technical step to get the analytical result for the Poisson case is well known, but the major technical step to get the analytical result for the Gaussian case is, to the best of my knowledge, a new contribution to the PSD literature. In general, the source that generates events for each class has a potentially broad energy deposit spectrum. For the Poisson case, for each event class, I assume knowledge of the Poisson parameters for a prompt time interval and a late time interval as a function of *e*. For the Gaussian case, for each event class, I assume knowledge of the mean and variance of the integrated pulse for both a prompt time interval and a late time interval as a function of *e*. For the cases studied, I assign an event to the signal class if the observed value of *X_p_* exceeds a selected discrimination threshold that in general depends on the observed value of *X_t_*. With a Bayesian method, I determine the posterior mean background acceptance probability as well as the posterior mean signal acceptance probability. My methods should facilitate evaluation of receiver-operating-characteristic curves (signal acceptance probability versus background acceptance probability) [35] for the Gaussian and Poisson cases.

## 2 Gaussian Noise Model

### 2.1 Conditional Distribution of *X_p_*

Throughout this work, I denote a random variable with a capital letter (e.g. *X*) and a particular realization of the random variable with a lower case letter (e.g. *x*). The prompt fraction statistic is a random variable $F_{p}=\frac{X_{p}}{X_{t}}$. I decompose *X_t_* into the sum of a prompt and late contribution, i.e.,

(3) *X_t_* = *X_p_*
+ *X_l_,*

where *X_p_* is the integrated pulse measured during [*T_begin_, T_prompt_* ], and *X_l_* is the integrated pulse measured during (*T_prompt_, T_end_* ]. Here, I assume that *X_p_* and *X_l_* are independent Gaussian random variables with known energy-dependent means *µ_p_*(*e*) and *µ_l_* (*e*), and known energy-dependent variances $\sigma_{p}^{2}(e)$and $\sigma_{l}^{2}(e)$. Given these assumptions, the expected value and variance of *X_t_* are

(4) *µ_t_* (*e*) =
*µ_p_*(*e*) +
*µ_l_* (*e*),

and


(5) $$\sigma_{t}^{2}(e)=\sigma_{\rho }^{2}(e)+\sigma_{l}^{2}(e).$$

Further, the correlation *ρ* between *X_p_* and *X_t_* is


(6) $$\text{ρ}(e)=\frac{E\left(\left(X_{p}-\mu_{\rho }\left(e\right)\right)\left(X_{t}-\mu_{t}\left(e\right)\right)\right)}{\sigma_{p}(e)\sigma_{t}(e)}=\frac{\sigma_{\rho }(e)}{\sigma_{t}(e)}.$$

As discussed in many references including Ref. [36], if two Gaussian random variables *X* and *Y* have correlation *ρ*, the distribution of the conditional value of *Y* given the observed value of *X*, (*Y |X* = *x*), is a Gaussian random variable with expected value


(7) $$E(Y|X=x)=E(Y)+\text{ρ}\frac{\sigma_{Y}}{\sigma_{X}}(x-E(X)),$$

and variance


(8) Var(*Y |X* = *x*) = (1
*− ρ*^2^)Var(*Y*).

Hence, for the mono-energetic case, given that the observed value of *X_t_* is *x_t_* and the scaled energy deposit is *e*, *X_p_* is a Gaussian random variable with expected value


(9) $$E\left(X_{p}|X_{t}=x_{t},E=e\right)=\mu_{p}(x_{t},e)=\mu_{p}(e)+\frac{\sigma_{p}^{2}(e)}{\sigma_{t}^{2}(e)}(x_{t}-\mu_{t}(e)),$$

and variance


(10) $$\text{Var}\left(X_{p}|X_{t}=t,E=e\right)=\sigma_{p}^{2}(x_{t},e)=\sigma_{\rho }^{2}(e)(1-\frac{\sigma_{\rho }^{2}(e)}{\sigma_{t}^{2}(e)}).$$

### 2.2 Acceptance Probabilities

Without loss of generality, I assume that events produced by the signal of interest yield, on average, larger observations of *X_p_* (compared to background events) for any particular scaled energy deposit *e*. Given this assumption, a natural classification rule is to assign an event to the signal class if the observed value of *X_p_* exceeds a discrimination threshold *c*(*x_t_*) that depends on the observed value of *X_t_* . In general, many scientific considerations influence the choice of the discrimination threshold *c*(*x_t_*). Given that *F*(*x,µ,σ*) is the cumulative distribution function (at *x*) for a Gaussian random variable with mean *µ* and standard deviation *σ*, the background acceptance probability, *p_BG_*(*x_t_,e*), is


(11) *p_BG_*(*x_t_,e*) =
1*−F*( *c*(*x_t_*),
*µ_p_*(*x_t_,e,B*),
*σ_p_*(*x_t_,e,B*) ),

where *µ_p_*(*x_t_,e,B*) is the Eq. (9) prediction of *µ_p_*(*x,e*) for the background class, and *σ_p_*(*x_t_,e,B*) is the Eq. (10) prediction of *σ_p_*(*x_t_,e*) for the background class. The signal acceptance probability is

(12) *p_S_*(*x_t_,e*)
= 1 *−F*( *c*(*x_t_*),
*µ_p_*(*x_t_,e,S*),
*σ_p_*(*x_t_,e,S*)),

where *µ_p_*(*x_t_,e,S*) and *σ_p_*(*x_t_,e,S*) correspond to the Eq. (9) and Eq. (10) predictions for the signal class.

#### 2.2.1 Posterior Means of Acceptance Probabilities

I account for uncertainty in the scaled energy deposit that produces any particular event with a Bayesian method. For a comprehensive review of Bayesian methods, see Ref. [37]. For the ideal case where one has an exact model for the scaled energy deposit spectrum due to background events, the prior distribution for the scaled energy deposit would be equated to this spectrum. However, in general, such an exact model may not be available. For the general case, the prior distribution would be selected by scientific judgement.

I denote the prior distribution for the scaled energy deposit due to a background event as *π_BG_*(*e*). For the Gaussian noise model, the conditional probability density function of *X_t_* given that *E* = *e* is


(13) $$f_{d}(X_{t}=x_{t}|e)=\frac{1}{\sqrt{2\pi }\sigma_{T}(e)}\exp(-\frac{(x_{t}-\mu_{T}(e){)}^{2}}{2\sigma_{T}^{2}(e)}).$$

Without loss of generality, I assume that *µ_T_* (*e*) and *σ_T_* (*e*) are the same for both the signal class and the background class. By Bayes’ theorem, the posterior distribution for *E* given *X_t_* = *x_t_* for a background event is


(14) $$f_{e}\left(e|x_{t}\right)=\frac{f_{d}\left(x_{t}|e\right)\pi_{BG}(e)}{\int_{e}\;f_{d}\left(x_{t}|e\right)\pi_{BG}(e)de}$$

Hence, given *x_t_*, the posterior mean of the acceptance probability for the background class is


(15) $${\bar{p}}_{BG}(x_{t})=\int_{e}p_{BG}(x_{t},e)f_{e}(e|x_{t})de.$$

By similar methods, one can derive the posterior mean of the acceptance probability for the signal class as


(16) $${\bar{p}}_{S}(x_{t})=\int_{e}p_{S}(x_{t},e)f_{e}(e|x_{t})de.$$

As a caveat, if the prior distribution for the scaled energy deposit for the signal class differs from *π_BG_*(*e*), *f_e_*(*e|x_t_*) in Eq. (16) would differ from the corresponding expression in Eq. (15).

### 2.3 Simulation Study

I assume that energies and integrated voltage pulses are dimensionless. As an illustrative example, I assume that *β* = 1 [see Eq. (2)] for both classes, and that


(17) $$\mu_{p}(e)=e(\alpha +\beta (1-\exp(-\frac{e}{200}))),$$

(18) $$\mu_{t}(e)=e,$$

and

(19) *µ_l_* (*e*) =
*e − µ_p_*(*e*).

**Fig. 1:**
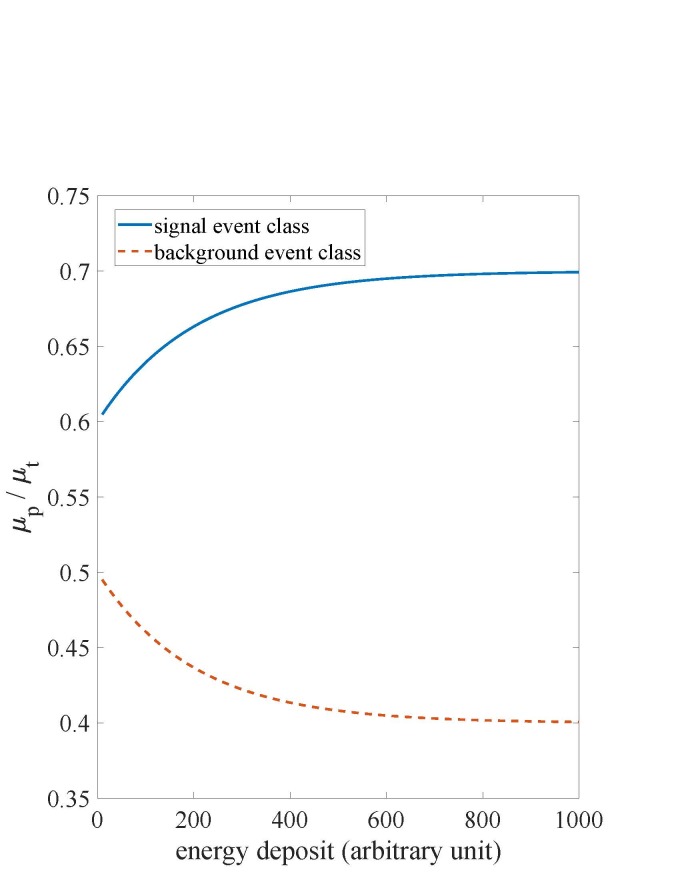
The theoretical prompt fraction, *µ_p_/µ_t_* , varies with the energy deposit for both classes in the simulation experiment. Because the relative yield term *β* equals 1 for both classes, the scaled energy deposit [see Eq. (2)] and energy deposit agree in this study.

For the signal class, (*α, β*) = (0.6,0.1). For the background class, (*α, β*) = (0.5,-0.1) (see Fig. 1). For both classes,

and

(20) $$\sigma_{l}^{2}(e)=2\mu_{l}(e)+1$$,

and

(21) $$\sigma_{p}^{2}(e)=2\mu_{p}(e)+1$$.

Given the observed value *x_t_*, I estimate *e* to be *ê* = *x_t_* . In the primary studies presented here, I set the discrimination threshold *c*(*x_t_*) to be the expected value of (*X_p_|X_t_* = *x_t_, E* = *ê*) for signal events [see Eq. (9)]. This choice corresponds to a target signal acceptance of 0.5. Since *µ_t_* (*e* = *x_t_*) = *x_t_*, *c*(*x_t_*) = *µ_p_*(*e* = *x_t_*).

**Fig. 2:**
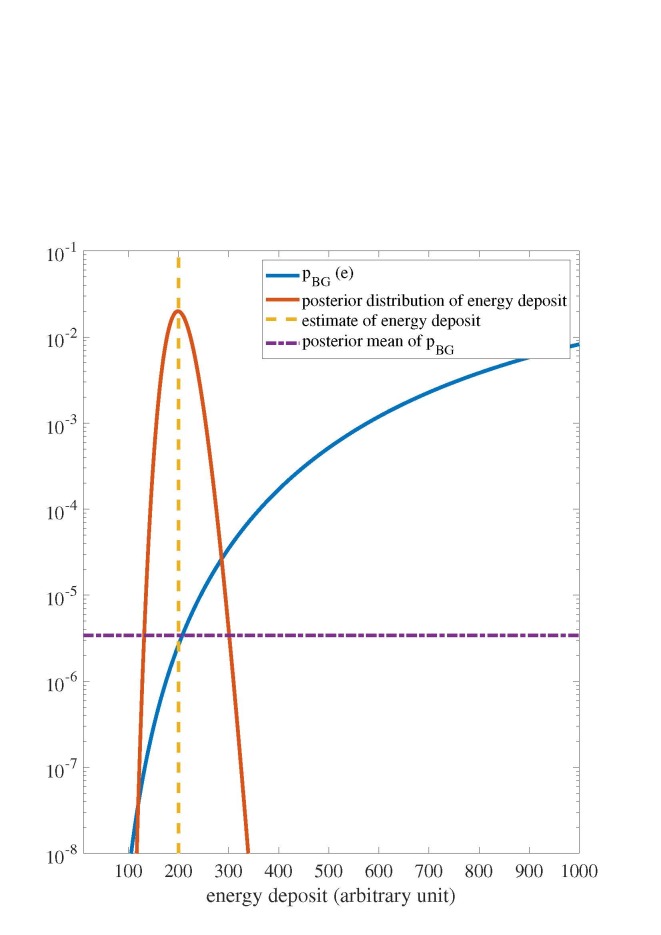
Gaussian noise model where *x_t_* = 200 and the discrimination threshold yields a target signal acceptance probability of 0.5. The posterior probability density function distribution of the energy deposit The posterior mean *${\bar{P}}_{\mathrm{BG}}$* derived from Eq. (15), is determined with a uniform prior distribution.

In Fig. 2, I illustrate my method for the case where *x_t_* = 200 and the prior distribution for *e*, *π_BG_*(*e*), is uniform for the range 10 *≤ e ≤* 1000. At other values of *e*, the prior distribution is 0. I also determine results for a truncated exponential prior distribution for the range 10 *≤ e ≤* 1000 where


(22) $$\pi_{\mathrm{BG}}(e)\propto \exp(-\frac{e}{500}).$$

At other values of *e*, the prior distribution is 0 (see Table 1).

**Table 1:** Gaussian noise model. Posterior mean of background acceptance probability and posterior mean of signal acceptance probability given that the target signal acceptance probability is 0.5. The exponential prior distribution is defined in Eq. (22).

$x_{t}$	${\bar{P}}_{\mathrm{BG}}(x_{t})$ exponential prior distribution	${\bar{P}}_{\mathrm{BG}}(x_{t})$ uniform prior distribution	${\bar{P}}_{S}(x_{t})$ exponential prior distribution	${\bar{P}}_{S}(x_{t})$ uniform prior distribution
100	6.11 x 10^-3^	6.17 x 10^-3^	0.508	0.512
200	3.31 x 10^-6^	3.40 x 10^-6^	0.504	0.511
300	2.47 x 10^-10^	2.62 x 10^-10^	0.500	0.509

### 2.4 Predicted Background Spectrum

In an actual experiment, one might wish to predict the background rate in each of many bins in *x_t_ −*space. For an experiment where the expected number of background events is E(*N_BG_*), the predicted number of background events that are assigned to the signal class for values of *x_t_* in the interval (*x_k_ −* ∆*/*2, *x_k_* + ∆*/*2) is E(*N_k_*), where


(23) $$E(N_{k})\;=\;E(N_{BG})\int_{e}\int_{x=x_{k}-\Delta /2}^{x_{k}+\Delta /2}\pi_{BG}(e)p_{BG}(x,e)f_{d}(x|e)dxde.$$

For very narrow bins in *x_t_* -space,


(24) $$E(N_{k})\;\approx \;E(N_{BG}){\bar{p}}_{BG}(x_{k})\Delta \int_{e}\pi_{BG}(e)f_{d}(x_{k}|e)de.$$

## 3 Poisson Noise Model

I assume that *X_p_* and *X_l_* are independent Poisson random variables. For the signal class, their Poisson parameters are *λ_p_*(*e,S*) and *λ_l_* (*e,S*). For the background class, their Poisson parameters are *λ_p_*(*e,B*) and *λ_l_* (*e,B*). Hence, the theoretical prompt ratios for the signal class and background class are

(25) $$r_{S}(e)=\frac{\lambda p(e,S)}{\lambda p(e,S)+\lambda_{l}(e,S)}$$,

and


(26) $$r_{\mathrm{BG}}(e)=\frac{\lambda p(e,B)}{\lambda p(e,B)+\lambda_{l}(e,B)}.$$

Given that *N*_1_ and *N*_2_ are independent Poisson random variables with Poisson parameters *λ*_1_ and *λ*_2_ and *N* = *N*_1_ + *N*_2_, the conditional distribution of *N*_1_, given that the observed value of *N* is *n*, is a binomial random variable with parameters *n* and *p* where *$p=\frac{\lambda_{1}}{\lambda_{1}+\lambda_{2}}.$* This well-known result follows from the following conditional probability equality:


(27) $$\mathrm{Pr}(N_{1}=k|N=n)=\frac{\mathrm{Pr}(N_{1}=k,N_{2}=n-k)}{\mathrm{Pr}(N=n)}=\frac{\mathrm{Pr}(N_{1}=k)\mathrm{Pr}(N_{2}=n-k)}{\mathrm{Pr}(N=n)}.$$

Thus, for the mono-energetic case, given that *X_t_* = *x_t_*, (*X_p_|X_t_* = *x_t_, E* = *e*) is a binomial random variable with parameters *x_t_* and *r_S_*(*e*) for the signal class. For the background class, (*X_p_|X_t_* = *x_t_, E* = *e*) is a binomial random variable with parameters *x_t_* and *r_BG_*(*e*).

Given that *G*(*k,N,p*) is the cumulative distribution function (at *k*) of a binomial random variable with parameters *N* and *p*, the acceptance probabilities for the background and signal classes are


(28) $$p_{BG}(x_{t},e)=1-G(c(x_{t}),x_{t},r_{BG}(x_{t})),$$

and


(29) $$p_{S}(x_{t},e)=1-G(c(x_{t}),x_{t},r_{S}(x_{t})).$$

By Bayes’ theorem, the posterior distribution for *E* given *X_t_* = *x_t_* is


(30) $$f_{e}(e|x_{t})=\frac{p_{d}(x_{t}|e)\pi_{BG}(e)}{\int_{e}p_{d}(x_{t}|e)\pi_{BG}(e)de},$$

where the conditional probability mass function of *X_t_* given that *E* = *e* is


(31) $$p_{d}(X_{t}=x_{t}|e)=\frac{\exp(\;-\lambda_{t}(e)\;)\;\lambda_{t}x^{t}(e)\;}{x_{t}!},$$

where *λ_t_* (*e*) is the expected value of (*X_t_ |E* = *e*). Based on Eqs. (28) to (31), the posterior means of the acceptance probabilities for the background class and signal class are then determined with Eq. (15) and Eq. (16).

**Fig. 3:**
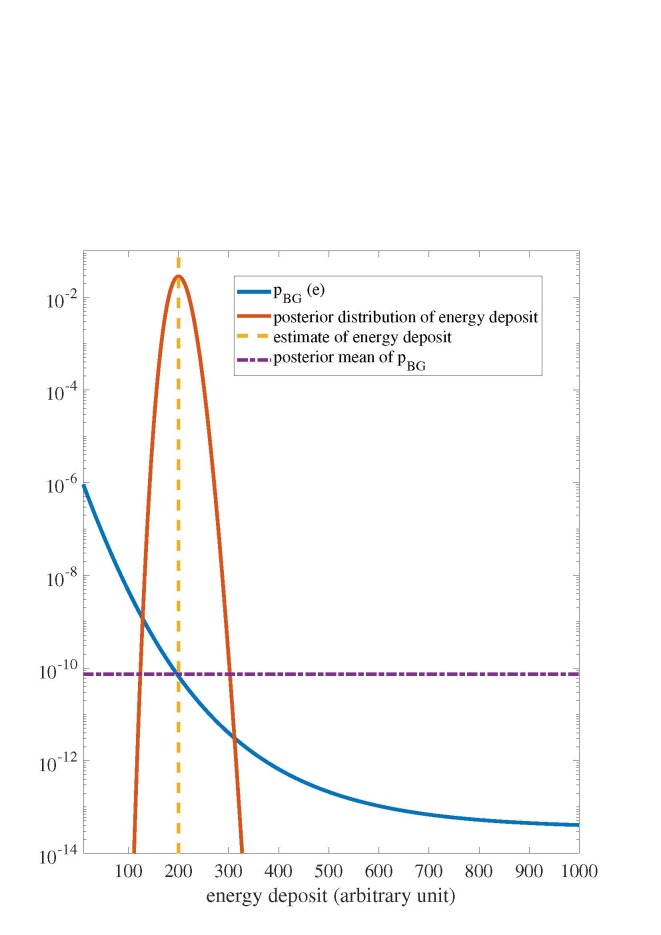
Poisson noise model where $x_{t}$ = 200 and the discrimination threshold yields a target signal acceptance probability of 0.5. The posterior mean ${\bar{P}}_{\mathrm{BG}}$ derived from Eq. (15), is determined with a uniform prior distribution.

In a simulation study, I determine posterior mean acceptance probabilities for the Poisson case (see Fig. 3; Table 2). In this study, I set *λ_p_*(*e,S*) and *λ_l_* (*e,S*) to the values of *µ_p_*(*e*) and *µ_l_* (*e*) assumed for the signal class in Sec. 2.3. I also set *λ_p_*(*e,B*) and *λ_l_* (*e,B*) to the values of *µ_p_*(*e*) and *µ_l_* (*e*) assumed for the background class in Sec. 2.3. I also set *λ_t_* (*e*) to the value of *µ_t_* (*e*) assumed in Sec. 2.3. I select a threshold corresponding to a target signal acceptance of 0.5. Given *x_t_*, this threshold is *c*(*x_t_*) = *x_t_r_S_*(*e* = *x_t_*).

**Table 2:** Poisson noise model. Posterior mean of background acceptance probability and posterior mean of signal acceptance probability given that the target signal acceptance probability is 0.5. The exponential prior distribution is defined in Eq. (22).

$x_{t}$	${\bar{P}}_{\mathrm{BG}}(x_{t})$ exponential prior distribution	${\bar{P}}_{\mathrm{BG}}(x_{t})$ uniform prior distribution	${\bar{P}}_{S}(x_{t})$ exponential prior distribution	${\bar{P}}_{S}(x_{t})$ uniform prior distribution
100	2.34 x 10^-4^	2.33 x 10^-4^	0.541	0.542
200	7.45 x 10^-11^	7.35 x 10^-11^	0.512	0.513
300	1.27 x 10^-34^	9.78 x 10^-35^	0.493	0.494

### 3.1 Receiver-Operating-Characteristic Curve

**Fig. 4:**
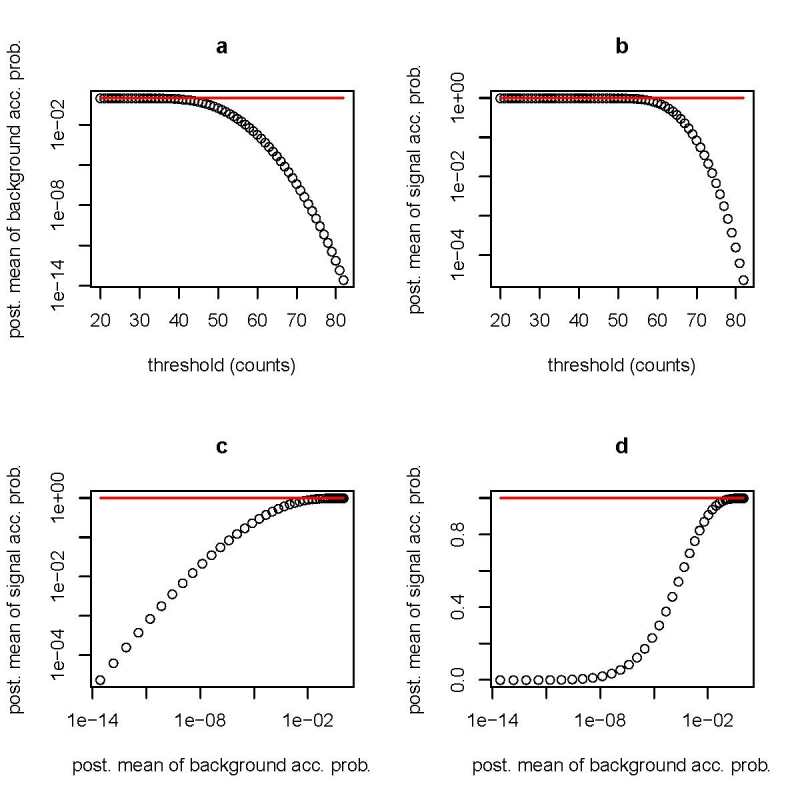
Poisson noise model where *x_t_* = 100. (a) Posterior (post.) mean of background acceptance probability (acc. prob.) $\left({\bar{P}}_{\mathrm{BG}}\right)$ versus discrimination threshold. (b) Posterior mean of signal acceptance probability (${\bar{P}}_{S}$) versus discrimination threshold. (c) ROC curve (*${\bar{P}}_{S}$* versus *${\bar{P}}_{\mathrm{bg}}$*) on log-log scale. (d) ROC curve on log-linear scale. Posterior means are determined with a uniform prior distribution. The horizontal line corresponds to 1.

In Fig. 4, I show how to construct a receiver-operating-characteristic (ROC) curve for any particular value of *x_t_* for the Poisson noise model. In this study, *x_t_* = 100 and the theoretical model for the Poisson parameters for the prompt and late time intervals for the signal and background are the same as discussed earlier. In Eq. (28) and Eq. (29), the discrimination threshold, *c*(*x_t_*), is varied over a broad range of integer values (20 to 83). For each candidate discrimination threshold, I determine the posterior mean value of *p_BG_*($x_{t}$,* e*) and the posterior mean value of *p_S_*($x_{t}$*, e*) (see Figs. 4a and 4b). Each candidate discrimination threshold yields a distinct value of $\left({\bar{P}}_{\mathrm{BG}}(x_{t}),({\bar{P}}_{S}(x_{t})\right)$. The ROC curve is the union of all distinct values of $\left({\bar{P}}_{\mathrm{BG}}(x_{t}),({\bar{P}}_{S}(x_{t})\right)$ (see Figs. 4c and 4d). One can construct an ROC curve for the Gaussian noise model with a similar approach.

## 4 Discussion

For both the Poisson and Gaussian models, for any particular energy deposit, I assume that *X_p_* and *X_l_* are independent random variables (see Sec. 2.1 and Sec. 3). As discussed earlier (see Sec. 1), in many experiments, only a fraction of the full energy deposit produces measurements of interest. As remarked earlier, I assume in this work that this fraction does not vary from event-to-event for each class. If this fraction randomly varies from event-to-event, I expect *X_p_* and *X_l_* to be positively correlated for any particular energy deposit. The models in this work do not account for this correlation structure.

In the simulations reported here, the posterior mean of the background acceptance probability increases as the energy deposit increases for the Gaussian model (see Fig. 2). In contrast, for the Poisson model, the posterior mean of the background acceptance probability decreases as the energy deposit increases (see Fig. 3). I attribute this result to the fact that the fractional standard deviation (standard deviation divided by expected value) of the conditional value of *X_p_* is larger for the Gaussian case relative to the Poisson case.

The choice of prior distribution affected results slightly (see Tables 1 and 2). As a caveat, there may be other prior distributions of interest.

## 5 Summary

In this theoretical study, I derived analytical expressions that quantify the performance of a ratio-based pulse shape discrimination method for Gaussian and Poisson noise models. With a Bayesian method, for a particular target acceptance probability for the signal class events, I determined the posterior mean background acceptance probability as a function of the observed value of *X_t_* in a way that accounted for imperfect knowledge of the energy deposit. In a simulation study, I determined results for two choices of the prior distribution in the Bayesian method (see Tables 1 and 2).

My analytic methods may enable one to determine receiver-operating-characteristic curves by numerical integration rather than by Monte Carlo simulation. My methods may provide experimentalists with useful theoretical predictions of ratio-based P performance in planning studies provided that integrated pulses are well approximated as realizations of either Gaussian random variables or Poisson random variables, and accurate models for the energy-dependent distributions of *X_p_* and *X_t_* are available for background events and signal events.
